# Dr. Davis’ History

**Published:** 1851-01

**Authors:** 


### ARTICLE III.

### DR. DAVIS' HISTORY.

An explanation for sending the extra pamphlet containing the concluding chapter of Prof. Davis' History should have been given in our last. Finding we could not publish the History in this volume of the Journal without crowding out much other matter, we thought it better to get the sheets from the book as it was going through the press, and in this manner complete the work. The size of this pamphlet is a little different from the Journal, but as it is paged to come at the back of this volume, it will bind with it very well. By this, we will give some 60 pages extra in this volume.

